# Imported Malaria in New York: Geographic Patterns and Implications for Emergency Physicians

**DOI:** 10.7759/cureus.91971

**Published:** 2025-09-10

**Authors:** Zachary Webb

**Affiliations:** 1 Emergency Medicine, Northwell Health, New Hyde Park, USA

**Keywords:** disease surveillance, emergency medicine, imported malaria, new york, public health

## Abstract

Although malaria is no longer endemic in the United States, imported cases continue to pose a diagnostic challenge for emergency physicians. This study examined county- and city-level trends in imported malaria across New York State (NYS) and New York City (NYC) from 2018 to 2024. Surveillance data revealed that NYC accounted for the majority of reported cases (79.1%). Within NYS, 11 of 57 counties (Albany, Dutchess, Erie, Monroe, Nassau, Oneida, Onondaga, Orange, Suffolk, Sullivan, and Westchester) contributed 88.3% of all state cases outside NYC. A marked decline in cases occurred in 2020, coinciding with COVID-19-related travel restrictions, followed by a rebound in 2021. These findings underscore the importance of incorporating both recent travel history and awareness of local county-level malaria cases into the evaluation of febrile patients in New York emergency departments, while also highlighting the need for targeted malaria prevention strategies in the state’s identified hotspots.

## Introduction

For emergency physicians in New York, malaria is a low-frequency but high-consequence diagnosis that can be easily missed until it becomes life-threatening. Caused by the *Plasmodium* species and transmitted by infected *Anopheles* mosquitoes, malaria may present in the emergency department (ED) as nothing more than fever, chills, myalgias, or headache, yet can progress rapidly to anemia, shock, coma, and death if untreated. Although preventable and curable, the disease remains endemic in much of Africa, Central and South America, the Caribbean, and Asia. In 2023, the World Health Organization (WHO) reported 263 million global cases, with more than half occurring in just five African nations [1]. In that same year, an estimated 597,000 people died from malaria worldwide [1].

Malaria was eliminated from the United States (U.S.) in 1951 [2]. Still, imported cases remain a recurring challenge. Epidemiological analyses of U.S. surveillance data show that New York had the highest average annual number of imported malaria cases (296.4 cases per year) between 2011 and 2016, leading the nation in disease burden [3]. More recently, in 2022, New York accounted for roughly 14% of all reported U.S. cases, the majority in New York City (NYC) [4]. The state’s disproportionate burden likely reflects its diverse immigrant population, multiple international airports, and the prevalence of travel among individuals visiting friends and relatives (VFRs). VFR travelers are particularly vulnerable: they represent the majority of imported malaria cases in the U.S., yet multiple studies show that more than 70% do not adhere to recommended chemoprophylaxis, placing them at markedly higher risk of infection [5,6].

For emergency physicians, the rarity of malaria in the U.S. can foster diagnostic complacency, yet for patients returning from endemic regions, a missed or delayed diagnosis can be fatal. The primary objective of this study is to quantify the geographic distribution patterns of imported malaria in New York between 2018 and 2024. The secondary objectives are to (1) identify high-incidence counties outside NYC and (2) assess the impact of the COVID-19 pandemic on case distribution. By linking geographic surveillance data with clinical practice, this study aims to guide targeted prevention strategies, enhance clinician readiness, and improve patient outcomes across New York EDs.

## Materials and methods

Imported malaria case data were obtained from three publicly available sources: (1) the New York State Department of Health’s (NYSDOH) Communicable Disease Annual Reports (2018-2023) [7], (2) the Statewide Mosquito-Borne Disease Activity Reports (2024) [8], and (3) the New York City Department of Health and Mental Hygiene’s (NYC DOHMH) Epi Data Briefs on Malaria (2013-2024) [9]. In this study, “New York State” (NYS) refers to all counties excluding the five boroughs of New York City (NYC).

The NYSDOH annual reports provided county-level counts for NYS and statewide totals, which included NYC as a single aggregate category. Annual NYC-specific case counts were obtained from the NYC DOHMH Epi Data Briefs. These reports provide citywide totals but do not list cases by individual borough, limiting the granularity of analysis within NYC. Because the NYSDOH annual reports extend only through 2023, statewide case numbers for 2024 were taken from the mosquito-borne disease activity reports. These reports use the same communicable disease surveillance definitions and case reporting procedures as the annual reports, minimizing inconsistency across years.

All datasets were consolidated into a single spreadsheet in Google Sheets (Google LLC, Mountain View, California, United States). Data cleaning involved verification of year-to-year totals, cross-checking overlapping reports, and confirming consistency across sources. No imputation was performed for missing data; counties without reported cases were classified as “zero reported cases.” Potential underreporting is acknowledged as a limitation of surveillance-based research.

For statistical analysis, data were exported into the DATAtab Statistics Calculator (DATAtab Team, 2025, DATAtab e.U. Graz, Austria). Annual case counts for NYC and NYS were summarized as means ± standard deviations. A Pearson correlation analysis was performed to assess the association between NYC and NYS annual case counts, with statistical significance set at p < 0.05. Given the small number of annual observations (n = 7 years), correlation results were interpreted with caution. A chi-square test was applied to evaluate whether malaria cases were uniformly distributed across NYS counties.

Ethical approval was not required, as only publicly available, de-identified data were analyzed.

## Results

NYC versus NYS case counts

Between 2018 and 2024, there were 1,919 reported imported cases of malaria in New York. Of these, 1,518 cases (79.1%) were reported from NYC and 401 cases (20.9%) from NYS.

**Figure 1:**
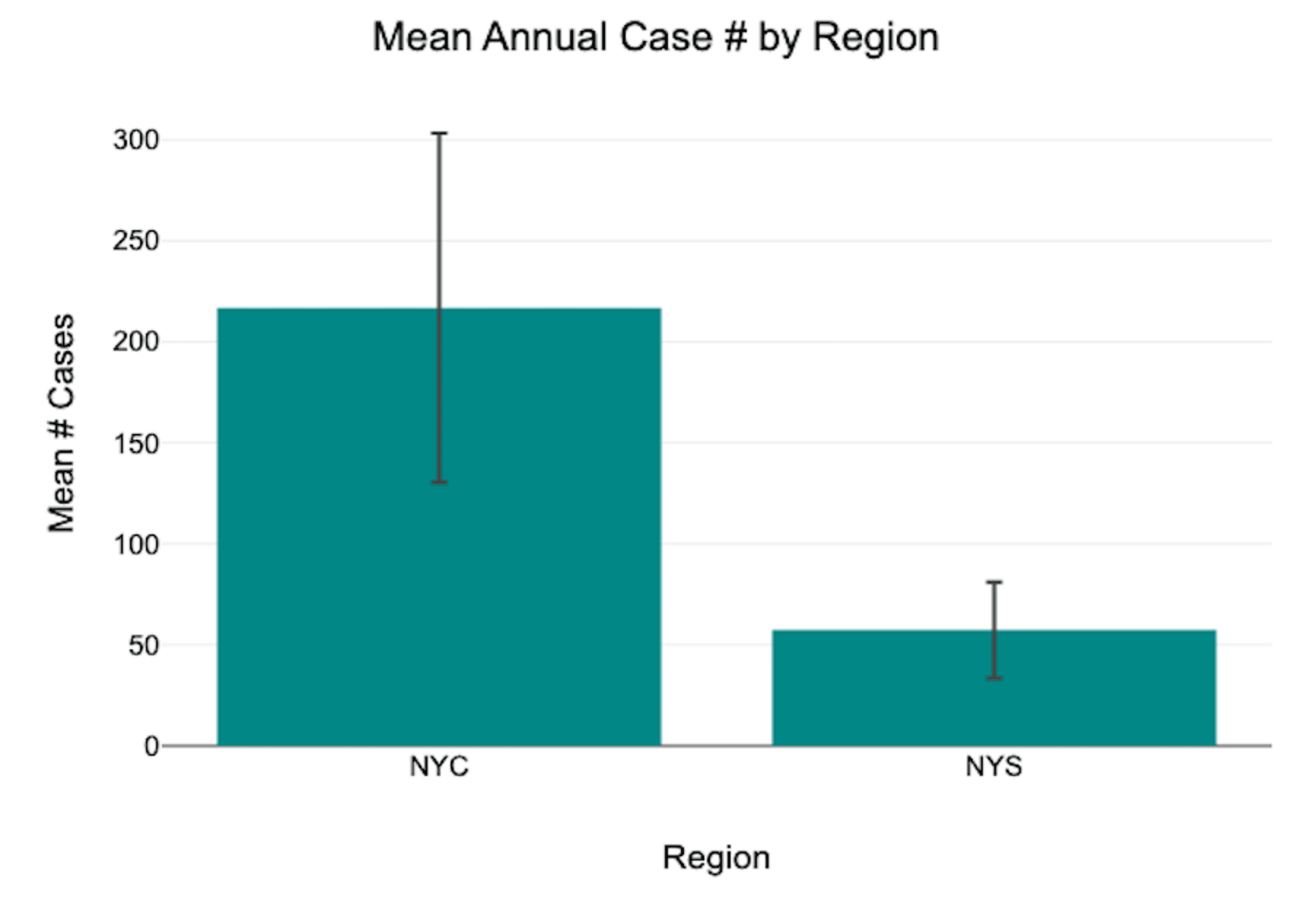
Annual imported malaria cases in NYC and NYS, 2018–2024. Data are presented as mean annual case counts ± standard deviation (mean ± SD). A Pearson correlation analysis demonstrated a statistically significant positive correlation between annual cases in NYC and NYS (r(5) = 0.96, p = 0.001; significance set at p < 0.05). NYS: New York State, NYC: New York City

In NYC, the mean annual case number was 216.9 ± 86.5, while in NYS, the mean annual case number was 57.3 ± 23.8 (mean ± SD; Figure 1). A Pearson correlation analysis demonstrated a statistically significant positive association between annual NYC and NYS case counts (r(5) = 0.96, p = 0.001). Given the small sample size (n = 7 years), this correlation should be interpreted with caution.

County-level distribution in NYS

**Figure 2:**
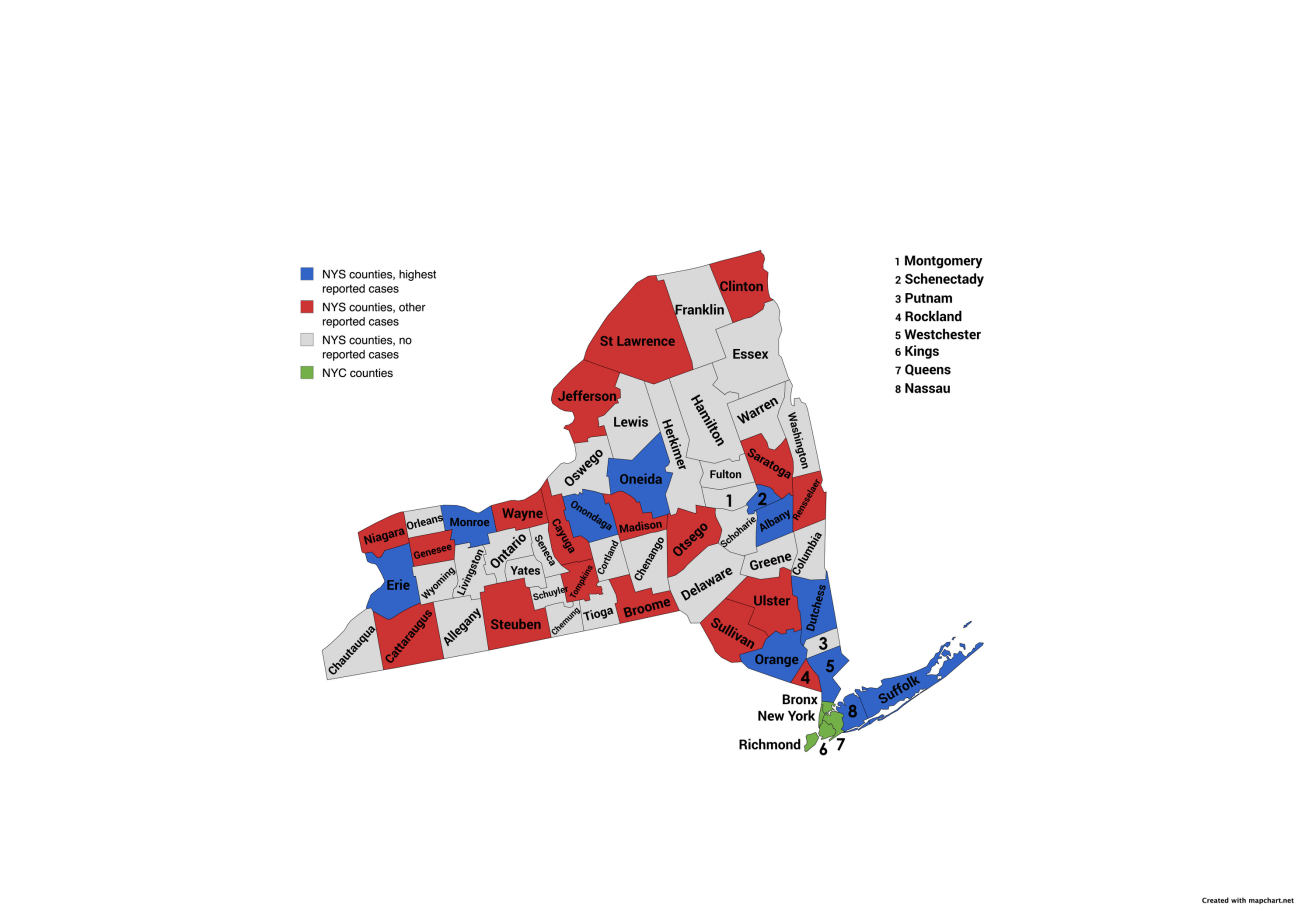
Spatial distribution of imported malaria cases by county in New York State, 2018–2024. Data are presented as a color-coded map by case count (N) category, distinguishing the 11 counties with the highest case counts, counties with other reported cases, counties with no reported cases, and NYC counties (shown separately). This figure is descriptive and does not include statistical testing; the study-wide significance threshold was p < 0.05. NYC: New York City

**Figure 3:**
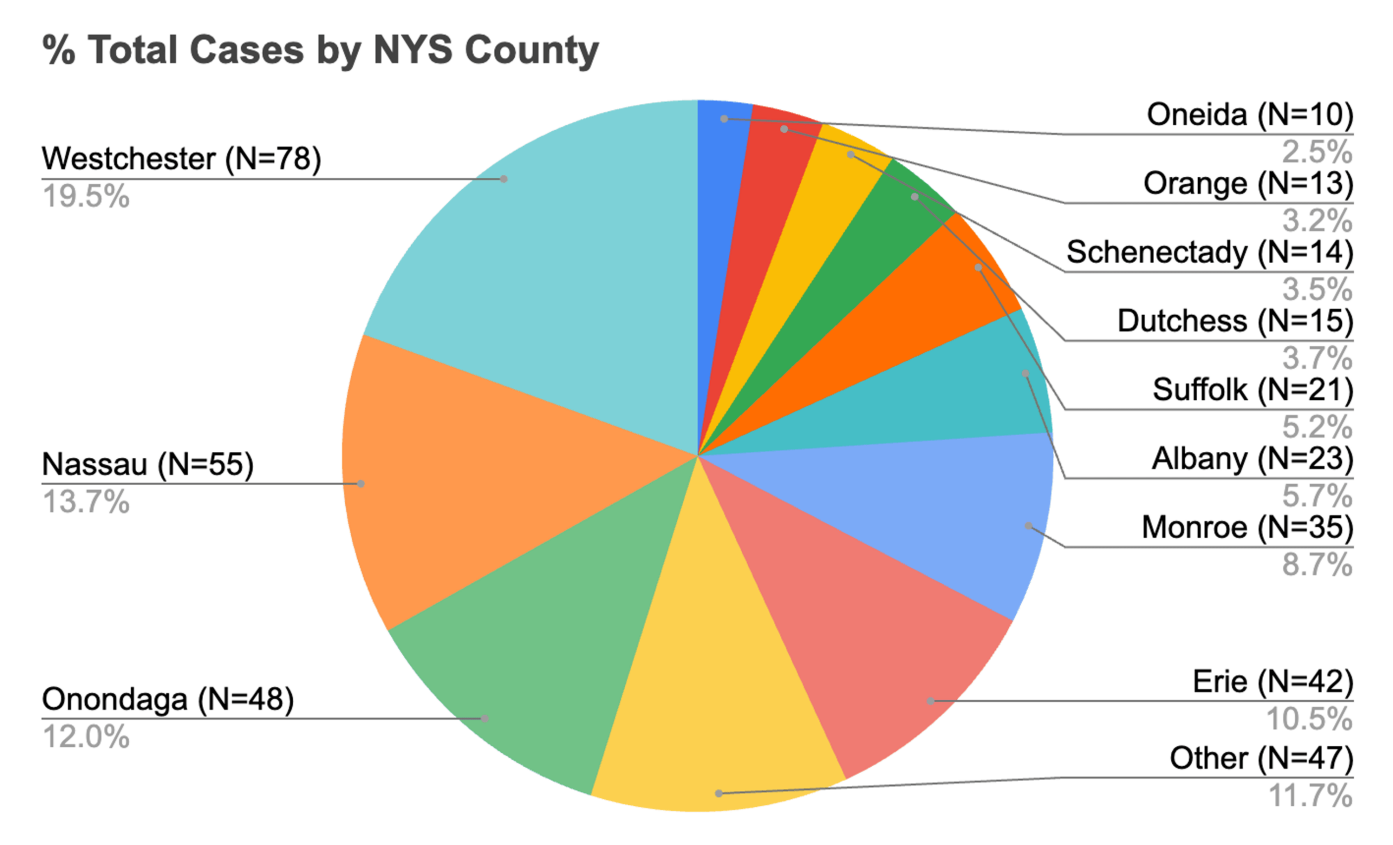
Spatial distribution of imported malaria cases by county in New York State, 2018–2024. Data are presented as a color-coded map highlighting the 11 counties with the highest number of reported imported malaria cases, counties with other reported cases, counties with no reported cases, and NYC counties (shown separately). A chi-square test demonstrated that case distribution was not uniform across NYS counties (χ²(56, N = 401) = 1910.98, p < 0.0001; significance set at p < 0.05). NYS: New York State, NYC: New York City

From 2018 to 2024, 29 of 57 counties (50.9%) reported at least one imported malaria case. Eleven counties - Albany, Dutchess, Erie, Monroe, Nassau, Oneida, Onondaga, Orange, Suffolk, Sullivan, and Westchester - accounted for 354 cases (88.3% of NYS total, 354/401) (Figures 2, 3). A chi-square test indicated that cases were not uniformly distributed across NYS counties (χ²(56, N = 401) = 1910.98, p < 0.0001).

Westchester County reported the highest number of cases (n = 78, 19.5% of NYS total). Its burden exceeded that of any other county, with case numbers peaking in 2021 (n = 13). The county’s consistently elevated incidence across multiple years distinguishes it as a geographic hotspot within NYS.

Temporal trends and COVID-19 impact

**Figure 4:**
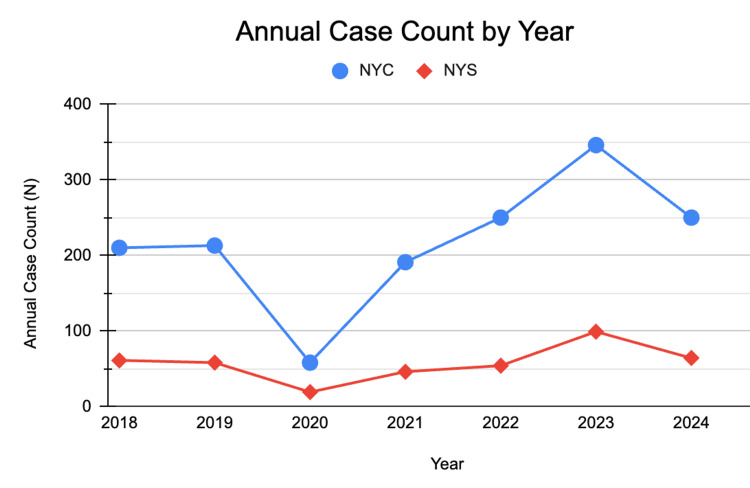
Annual number of reported imported malaria cases in NYC and NYS, 2018–2024. Line graph illustrating the total number of imported malaria cases reported annually in NYC (blue) and NYS (red). Data are presented as raw case counts (N) by year. This figure is descriptive and does not include statistical testing; comparative analyses are reported in the text. NYS: New York State, NYC: New York City

A secondary analysis evaluated the impact of the COVID-19 pandemic on imported malaria cases (Figure 4). There were 271 cases in 2019, including 213 cases in NYC (78.6%) and 58 cases in NYS (21.4%). In 2020, the total cases dropped sharply to 77, with 58 in NYC (75.3%) and 19 in NYS (24.7%). This represented a 71.6% overall reduction compared with 2019, including a 72.8% decline in NYC and a 67.2% decline in NYS.

In 2021, case numbers rebounded to 237, with 191 in NYC (80.6%) and 46 in NYS (19.4%). This represented a 207.8% increase compared with 2020, corresponding to a 3.3-fold increase in NYC and a 2.4-fold increase in NYS.

Westchester County accounted for a disproportionate share of NYS cases in 2021, rising from one case in 2020 to 13 cases in 2021 (28.3% of NYS total).

## Discussion

For emergency physicians in New York, imported malaria represents a rare but potentially fatal diagnosis that demands early recognition. This statewide analysis demonstrates that the majority of cases between 2018 and 2024 occurred in NYC, yet a substantial minority were consistently reported in select counties across the state. These findings underscore that malaria risk is not confined to NYC, and EDs across New York should maintain preparedness to evaluate febrile patients with compatible travel or exposure histories. Practical measures include maintaining rapid diagnostic capabilities and ensuring that malaria testing protocols are accessible in both urban and community hospitals.

While NYC accounted for nearly four out of every five reported cases, 11 counties outside the city contributed 88.3% of all NYS cases, indicating that imported malaria was concentrated in defined regions rather than evenly distributed across the state. These concentrations may reflect proximity to international travel hubs or other community-level factors, although such associations could not be confirmed with the available data. Westchester County stood out as the highest-burden county, responsible for nearly one-fifth of all NYS cases, underscoring the importance of recognizing county-level hotspots where EDs may encounter diagnostic challenges despite being outside the city’s highest-volume academic centers.

Temporal analysis revealed a sharp decline in malaria cases in 2020, with a 71.6% reduction compared with 2019, followed by a rebound of more than 200% in 2021. Although case counts in 2021 increased substantially compared with 2020, they remained 12.5% lower than 2019 levels. These shifts likely reflect changes in international mobility during the COVID-19 pandemic, underscoring how travel patterns can influence the burden of imported disease in local EDs. For clinicians, this highlights the importance of adjusting diagnostic suspicion in tandem with changes in travel patterns. Hospital networks may benefit from monitoring global travel trends and aligning diagnostic readiness accordingly.

From a prevention standpoint, adherence to chemoprophylaxis among at-risk populations remains a major gap. National data show that more than 70% of U.S. travelers diagnosed with malaria had not taken recommended prophylaxis [5], and VFR travelers are particularly vulnerable [6]. Targeted education and improved access to antimalarial prophylaxis, especially in counties identified as hotspots, may help reduce the future burden of imported malaria. ED encounters also represent an opportunity for clinicians to reinforce the importance of prophylaxis during discharge counseling of patients planning international travel.

This study has several important limitations. Surveillance data are subject to underreporting, delayed reporting, and variable sensitivity across counties and years. Borough-specific data were not available for NYC, preventing detailed intra-city analysis. Demographic and clinical variables, including patient ethnicity, reason for travel, adherence to chemoprophylaxis, and clinical outcomes, were unavailable, limiting the ability to validate hypothesized associations or assess diagnostic delays. The small number of annual observations (n = 7) reduces statistical power for trend analysis, and findings from correlation testing should be interpreted cautiously. Finally, the use of different state data sources for 2024 introduces minor temporal inconsistency, although both rely on the same surveillance case definitions.

Despite these limitations, the study provides practical, actionable insights. Malaria remains primarily concentrated in NYC but also demonstrates consistent clustering in several upstate and suburban counties. For emergency physicians, this reinforces the need to include malaria in the differential diagnosis of febrile patients throughout New York, not only in the city. Hospital systems and public health authorities should consider targeted prevention efforts, rapid diagnostic availability, and improved surveillance granularity to better prepare EDs for this ongoing challenge.

## Conclusions

For emergency physicians in New York, imported malaria remains an ongoing public health and clinical challenge. Between 2018 and 2024, most cases were reported in NYC, but nearly one-fifth occurred in select high-incidence counties across the state. These geographic hotspots may share features such as proximity to international travel hubs and other community-level factors, which could contribute to the elevated number of malaria cases observed in these areas, although these associations could not be confirmed with the available data.

For clinicians, the key implication is the continued importance of obtaining a thorough travel history and maintaining malaria in the differential diagnosis of febrile patients. Hospital networks and public health systems should ensure rapid access to malaria diagnostics in EDs statewide, with particular attention to NYC and the identified high-incidence counties.

Future efforts should focus on improving chemoprophylaxis adherence among at-risk travelers, strengthening surveillance systems with more granular demographic and clinical data, and monitoring global travel patterns to anticipate local diagnostic needs. These strategies may help reduce the burden of imported malaria and improve patient outcomes in New York EDs.
